# Setting the Standard for Peer‐Reviewed Published Studies on Regenerative Products in Aesthetic Medicine and Post‐Procedure Wound Care

**DOI:** 10.1111/jocd.70877

**Published:** 2026-05-06

**Authors:** Michael H. Gold, Terrence C. Keaney, Rebecca Fitzgerald

**Affiliations:** ^1^ Gold Skin Care Center Nashville Tennessee USA; ^2^ Tennessee Clinical Research Center Nashville Tennessee USA; ^3^ SkinDC, Clinical Associate Professor of Dermatology George Washington Hospital Arlington USA; ^4^ Private Practice Los Angeles California USA

The aesthetics market has historically been characterized by minimal regulatory oversight, leading to a proliferation of products with inconsistent safety testing data. The field of regenerative aesthetics, in particular, is relatively new and rapidly emerging. While regenerative aesthetics appears to hold great promise, and indeed may well include many of the most important advancements in our field, the growing and seemingly overwhelming number of products available that claim regenerative capacity can make it daunting for providers and their clients to sort out fact from hype and choose treatments backed by strong scientifically sound peer‐reviewed published evidence of their safe and effective use to achieve youthful, glowing skin and reduce post‐procedure complications and downtime.

In this context, two recent peer‐reviewed published articles titled “Safety of Exogenous Recombinant Human Platelet‐Derived Growth Factor‐BB (rhPDGF‐BB) for Medical and Cosmetic Applications: A Review” [1] and “Safety of up to 140 Daily Applications of Recombinant Human Platelet‐Derived Growth Factor (rhPDGF‐BB) onto Skin Wounds: Unboxing the Evidence” [2] are worthy of highlighting. These papers summarize an extensive portfolio of safety studies from both nonclinical models and randomized‐controlled clinical trials that have established the strong safety profile for recombinant pure PDGF‐BB manufactured and tested according to strict guidelines established by the Food and Drug Administration (FDA). The tens of thousands of study subjects, decades of follow‐up, rigorous scientific methods and statistical analyses applied and summarized in these articles must be recognized by our field as the standard for all regenerative products in dermatology, plastic and reconstructive surgery, aesthetic medicine and wound care.

Bioregenerative agents, like pure PDGF‐BB, have become increasingly popular in aesthetics due to the natural improvement in skin quality, texture, firmness, and radiance over time, rather than the immediate and often temporary physical augmentation of tissue provided by fillers. Unlike the more artificial effects of early aesthetic treatments, bioregenerative products stimulate the body's own healing and regenerative mechanisms at the cellular level, promoting long‐lasting structural and functional changes within the tissue, leading to sustained improvement over time and better results.

However, it is important for health care providers to be aware that not all purported regenerative products in aesthetic medicine have undergone rigorous nonclinical and clinical pharmacokinetic and toxicology studies to establish the margin of safety, potential risks, and contraindications. Pure PDGF‐BB presents a unique entry into aesthetic medicine compared to these other therapies. PDGF harnesses the body's innate healing power to regenerate and rejuvenate soft tissue and skin via stimulation of extracellular matrices such as collagen, elastin, and natural hyaluronic acid production and cellular renewal [3]. As the active pharmaceutical ingredient in four FDA‐approved products to treat chronic skin wounds and regenerate soft tissues and bone, pure recombinant PDGF‐BB research spans three decades and includes a robust portfolio of safety and pharmacology studies from standardized nonclinical models and long‐term randomized clinical trials [4].

As summarized in the two articles cited above, safety studies on recombinant pure PDGF‐BB include topical, injectable, and implantable routes of administration, across a wide range of doses—often thousands of times higher than we use in our field—and frequency—one time to weekly to daily applications—to establish the margin of safety and risks associated with its use [1]. Pharmacokinetic studies have demonstrated that exogenously applied PDGF‐BB acts locally, with a short half‐life and minimal systemic exposure even at very high doses in both animals and humans [5, 6, 7]. Skin irritation, hypersensitivity, and toxicity testing have revealed no significant adverse reactions to pure PDGF‐BB delivery via subcutaneous, intramuscular, intradermal, intraperitoneal, or intravenous injections, repeated topical administration, or implanted combination devices across a range of doses in multiple animal models [8, 9]. Reproductive toxicity studies reported no treatment‐related mortality or significant adverse effects in rats receiving daily IV injections of pure PDGF over 21 days of gestation [8, 9].

The clinical safety data for pure PDGF‐BB is even more impressive. The role of exogenous pure PDGF in cancer biology has been extensively studied, debated, and eventually refuted when the FDA removed the boxed warning after long‐term, retrospective, propensity‐matched studies comparing over 13 000 patients treated with up to 140 daily applications of topical PDGF‐BB or not treated with topical PDGF, and with over a decade of follow‐up showed no increased risk of cancer incidence or cancer‐related mortality with pure PDGF‐BB treatment [2, 10, 11]. Moreover, safety outcomes from over 100 clinical trials spanning 25+ years and involving over 15 000 subjects showed similar rates of adverse events and complications between PDGF‐treated and control groups in randomized‐controlled, double‐blinded clinical trials [4].

In summary, robust, scientifically sound safety data builds consumer trust in our field and gives providers confidence when making clinical decisions that will optimize patient outcomes. The extensive database of published studies on pure PDGF‐BB stands out as exemplary, rigorous testing of a regenerative product. The rigorous safety evaluations performed and evidence‐based data for pure PDGF‐BB summarized in these peer‐reviewed publications [1, 2] are a gift to our field and establish the standard that our profession should require for peer‐reviewed published studies before widespread commercial adoption of future bioregenerative products.

## Conflicts of Interest

The authors declare no conflicts of interest.

## Data Availability

The data that support the findings of this study are available from the corresponding author upon reasonable request.
